# Unraveling the Challenges: A Compelling Case of Staph Meningitis and Graft Infection in a Bovine Brain Graft Recipient

**DOI:** 10.7759/cureus.64806

**Published:** 2024-07-18

**Authors:** Sultan Ahmed, Ayeza Jamil, Moamen Al Zoubi

**Affiliations:** 1 Internal Medicine, Mercyhealth Hospital, Rockford, USA; 2 Internal Medicine, Allegheny Health Network, Erie, USA; 3 Internal Medicine, Lake Erie College of Osteopathic Medicine, Erie, USA; 4 Infectious Diseases, Mercyhealth Hospital, Rockford, USA

**Keywords:** s aureus, chiari malformation, bovine, graft infection, meningitis

## Abstract

Meningitis due to *Staphylococcus aureus* is extremely rare, with an annual incidence of 1-3%. In this report, we present a rare case involving meningitis, an infected graft, and an infected fluid collection with two forms of *S. aureus* in a patient who received a bovine brain graft status post-decompression and suboccipital craniectomy with C1 laminectomy and duraplasty for Chiari malformation. The treatment approach included surgical debridement and graft retention, followed by an extended course of antibiotic treatment with oxacillin and rifampin. The patient successfully completed 12 weeks of total antibiotic therapy and was transitioned to suppressive therapy indefinitely with cefadroxil. This case highlights the importance of prompt identification and treatment of *S. aureus* meningitis due to the high mortality associated with this disease.

## Introduction

Meningitis, which is defined as inflammation of the membranes of the skull and vertebral canal, has a high mortality rate of 25% [1]. It is often bacterial or viral in etiology, with symptoms including fever, neck pain/stiffness, and light sensitivity. Early management and treatment are essential, given the high mortality associated with meningitis. *Staphylococcus aureus* (*S. aureus*) meningitis is most commonly associated with recent surgery, central lines, or trauma [1]. It carries a significant mortality rate, with the mainstay treatment including nafcillin for methicillin-sensitive *S. aureus* (MSSA) and vancomycin for methicillin-resistant *S. aureus* (MRSA). Rifampin is considered as adjunctive therapy to vancomycin for severe cases of meningitis [2]. We describe a rare case of MRSA/MSSA meningitis in the setting of a recently placed brain graft. Our case highlights the importance of prompt recognition and treatment of MRSA/MSSA meningitis due to the high morbidity associated with this disease.

## Case presentation

A 33-year-old female with a past medical history of Chiari malformation, who recently underwent decompression including suboccipital craniectomy, C1 laminectomy, and duraplasty with a bovine patch, presented to the ED with the chief complaint of purulent drainage arising from the surgical site on her neck. The patient was discharged after her surgery and was overall doing well one week post-operatively. She subsequently developed two weeks of clear drainage with intermittent headaches and confusion and was started on cephalexin 500 mg twice daily for 10 days per neurosurgery. The patient also reported intermittent chills along with nausea and vomiting. She called the clinic with concerns of milky white drainage and was instructed to present to the ED for further evaluation.

**Figure 1:**
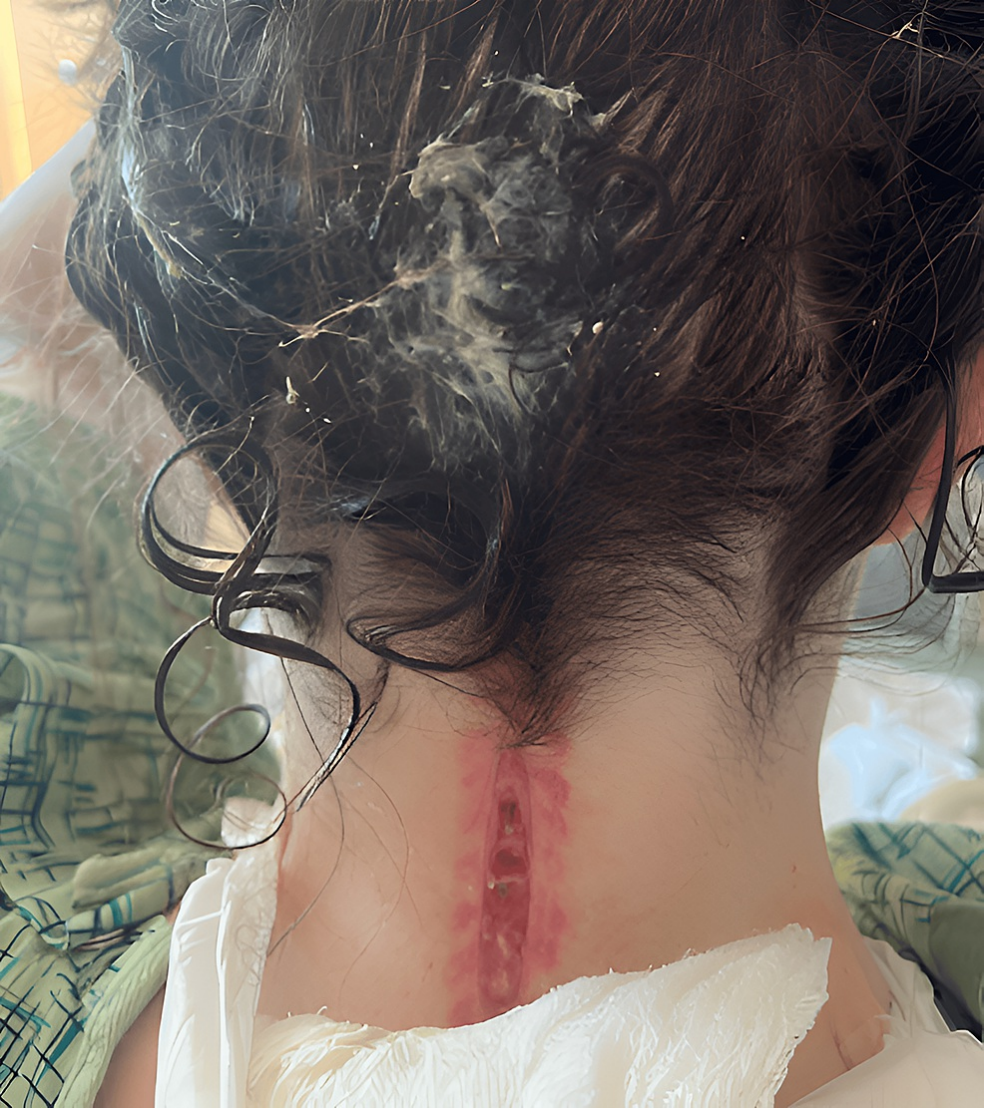
Image of the incision with purulent drainage on the patient’s initial arrival to the ER.

**Figure 2:**
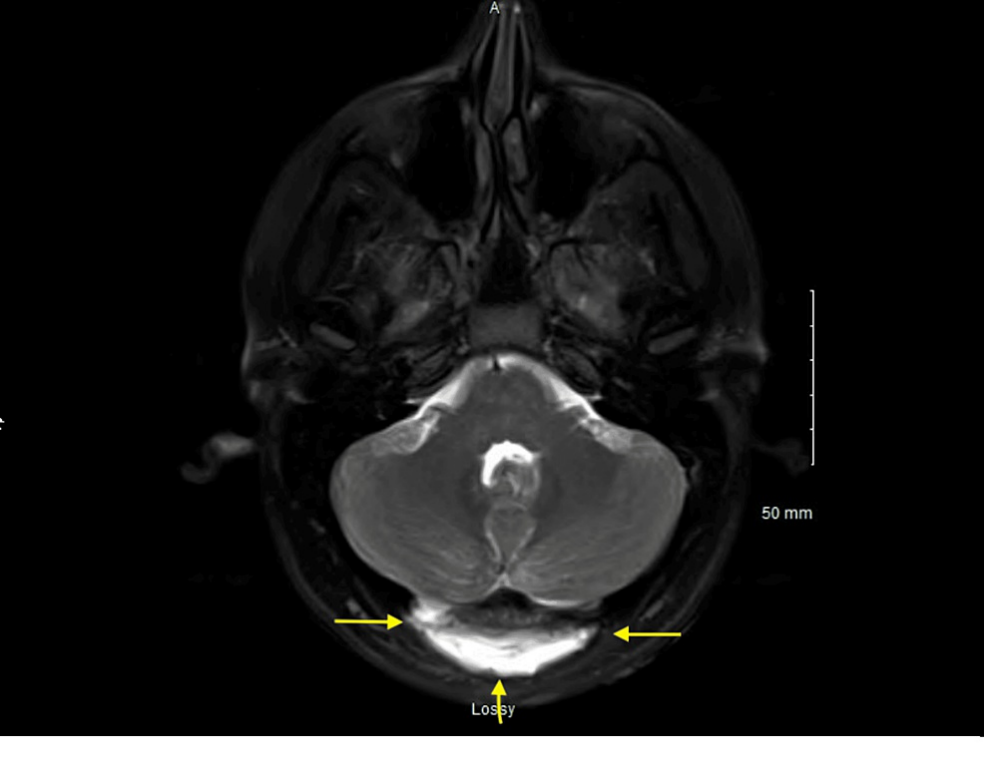
MRI performed on the patient’s initial arrival to the emergency room, demonstrating a complex fluid collection in the dorsal soft tissues measuring 1 x 6 x 7.5 cm.

In the ED, the patient was afebrile with stable vital signs. She had an elevated WBC count of 23,000 and was found to have purulent drainage from her incision site without erythema or warmth (Figure 1). She underwent MRI of the brain, lumbar puncture (LP), and MRSA swab. The patient was started on cefepime 2g IV every eight hours, vancomycin per pharmacy protocol, acyclovir 10 mg/kg every eight hours, and dexamethasone 0.15 mg/kg every six hours for presumed meningitis coverage while test results were pending. The MRI was significant for Chiari 1 malformation status post suboccipital decompression and complex fluid collection measuring 1 x 6 x 7.5 cm in the dorsal soft tissue, which could be correlated with a superimposed infection or abscess (Figure 2), and the MRSA swab was positive. The wound culture from the ER visit grew MRSA and MSSA. The LP resulted in a cell count of 1238 with 76% neutrophils, glucose of 44, and protein of 78. The Gram stain and anaerobic culture were negative, and the aerobic culture from cerebrospinal fluid (CSF) grew MSSA. Vancomycin and cefepime were continued per infectious disease recommendations, while acyclovir and dexamethasone were discontinued after one day of treatment in the setting of a bacterial infection. The patient underwent lumbar CSF drain placement with sharp wound debridement via neurosurgery to address the possible abscess collection versus a CSF leak identified on MRI. The patient's intraoperative wound specimen showed growth of MSSA.

CSF drainage via the catheter was analyzed and showed a cell count of 1192, 66% neutrophils, glucose of 29, and protein of 94. Cultures from the CSF were negative at 48 hours and held for 10 days to ensure no growth. Her antibiotic regimen was adjusted for better MSSA targeting; thus, cefepime was replaced by oxacillin 12 mg IV.

During the course of the hospital stay, the lumbar drain was accidentally removed; however, given no evidence of an external CSF leak, the neurosurgery team recommended no additional drain placement. Given her presentation and culture results, there was concern for bacterial biofilm formation on the bovine graft. The need for proper source control was discussed with the family; however, the consideration to remove the bovine pericardium patch graft for source control was not recommended per neurosurgery due to potential harm from removing the graft from the duraplasty and subsequent exposure of the posterior fossa and cerebellum.

In addition to adding oxacillin, IV rifampin 600 mg daily was added as studies have shown synergistic effects of oxacillin/nafcillin with rifampin in the setting of MSSA meningitis. Liver function tests were monitored daily while on rifampin. Oxacillin was continued at a dose of 2g IV every four hours. C-reactive protein was ordered daily during the duration of treatment, which was high at 22.70 mg/L and subsequently decreased with values of 18.98, 10.89, 9.0, and 8.75. The CRP trend was discontinued after consistent improvement was noted. After making changes to her antibiotic management, her WBC count was noted to be within normal limits at 12,800.

A peripherally inserted central catheter (PICC) line was placed for an extended duration of IV antibiotics post-discharge. Per infectious disease recommendation, the plan for antibiotic therapy included two weeks of IV vancomycin, and 10 weeks of IV oxacillin (this included days of antibiotics received while in-patient). Rifampin was switched to oral pills upon discharge. Post-PICC placement, the patient had drainage of CSF arising from her incision site, concerning for a CSF leak. This led to the reinsertion of a lumbar CSF drain per neurosurgery. CSF from lumbar drain reinsertion showed 98 WBC, 80% neutrophils, glucose of 34, and protein of 72. Cultures from the fluid were negative, and the gram stain showed no growth.

Post lumbar drain removal, the patient was discharged in stable condition with a plan for oxacillin 12 g continuous pump infusion daily and rifampin 300 mg per oral twice daily, for a total of 10 weeks of antibiotics directed at MSSA, in order to achieve penetration of the potential patch biofilm. In her outpatient follow-up after approximately five weeks, her physical exam showed a well-healed incision with no signs of infection. The patient had successfully completed 12 weeks of total antibiotic therapy, including the medications she was originally treated with prior to de-escalation for MSSA, and was transitioned to suppressive therapy with cefadroxil 500 mg per oral twice daily indefinitely.

## Discussion

Several points of benefit can be drawn from this case. Reviewed below are the guidelines for the treatment of *S. aureus* meningitis and points of consideration when managing such cases. We discuss a common defense mechanism employed by bacteria such as *S. aureus* to resist treatment, known as biofilms, the recommended treatments, adjuncts to the regimen such as rifampin or cephalosporins, and the consideration of using suppressive antibiotics for patients in remission after completion of curative therapy.

To begin, bacteria such as *S. aureus* and Staphylococcus non-aureus are commonly known to create biofilms; a system of survival often seen on medical implants and indwelling devices. A biofilm is a complex extracellular matrix and a biological system that bacteria develop to handle stressors and dynamic changes in their environments [3]. *S. aureus* is a major cause of community-acquired and nosocomial infections. Especially in the hospital setting, the formation of a biofilm that encases the bacterial cells leads to more chronic and harder-to-treat infections due to the resilience the biofilm provides against antibiotics. When colonized on human tissue in a wound or perhaps in bone, the host immune system can attack the biofilm, causing tissue damage and promoting further development of the bacterial microbiome [4]. In the case of prostheses, the approach to treatment involves the removal of the infected device along with prolonged antibiotic treatment. If a wound is involved, there is often prolonged antibiotic treatment with source control via debridement or perhaps amputation in the case of osteomyelitis.

To further characterize the pattern of biofilm formation by *S. aureus*, a study was performed by Silva V et al., which considered the ability of animal-strain *S. aureus* to create biofilms. They compared 214 strains of *S. aureus* isolated from pets, livestock, and wild animals, and it was found that many of these strains also had the ability to form biofilms. Stronger biofilms were associated with multi-drug resistant strains, and the use of tetracycline and amikacin at minimal inhibitory concentration (MIC) or 10 x MIC was not sufficient to eradicate some of these strains, showcasing high resistance to antibiotic treatment [5]. Bearing these mechanisms in mind, the Infectious Diseases Society of America (IDSA) has published a roadmap for managing bacteria such as *S. aureus*.

According to the IDSA, current guidelines for MSSA meningitis include nafcillin/oxacillin as the first-line agent. If a patient is allergic to these drugs, they can be desensitized or given vancomycin. The timeline for management can vary based on presenting factors such as CSF findings and patient presentation [6]. Rifampin can be used as an adjunct to the recommended antibiotic regimen. Rifampin achieves bactericidal concentrations in the CSF; some studies have shown that the concentration of rifampin can be higher in the CSF than vancomycin [7]. Use of rifampin has been shown to reduce CSF inflammation and bacterial count. This adjunct is recommended for patients with intracranial or spinal hardware such as a CSF shunt or drain [8]. However, to avoid rifampin resistance, it is recommended to initiate this antibiotic only after wounds are dry, drains have been removed, and at least 3-5 days of IV antibiotics have been administered to secure adequate bacterial load reduction [8]. Considering the role of cephalosporins, their impact on CSF infections varies greatly due to their ability to cross the blood-brain barrier. First- and second-generation cephalosporins, except for cefuroxime, are unable to reach high CSF concentrations; however, this medication is typically not used due to its adverse effects. Cefadroxil, a first-generation cephalosporin, has been shown to achieve distribution into the brain's extracellular and intracellular fluids when combined with probenecid [9]. Ceftriaxone, cefotaxime, ceftazidime, cefixime, and cefepime have been studied in children and are all able to adequately penetrate the CSF.

When considering MRSA meningitis, the IDSA recommends two weeks of IV antibiotics. The United Kingdom released guidelines in 2021 regarding MRSA meningitis treatment to include rifampin in conjunction with IV vancomycin for severe infections. If a patient failed to respond to IV vancomycin, it was recommended that the patient be transferred to a neurosurgical center to have vancomycin injected directly into the ventricles [10]. They did not recommend clindamycin, chloramphenicol, or linezolid to treat meningitis caused by MRSA as these drugs are not bactericidal. In the case of this patient, CSF culture did not grow MRSA, but wound culture did; therefore, she received two weeks of IV vancomycin.

Considering long-term management of patients, research has been performed looking into the benefits of long-term antibiotic suppressive therapy in the setting of foreign body retention, such as prostheses. For patients who are not deemed optimal candidates for foreign body removal or replacement, antibiotic suppressive therapy was evaluated [11]. Per the IDSA, doxycycline has been recommended in the past for suppressive therapy. In a study performed by Pradier M et al., doxycycline was used for patients demonstrating remission after antibiotic therapy but who were at high risk of the return of infection and not optimal candidates for surgical removal of the foreign body. The results of the study found that the use of doxycycline was associated with a good rate of remission and a low risk of bacterial resistance in the case of management failure [11]. In a study performed by Wouthuyzen-Bakker M et al., antibiotic suppressive therapy in patients with prosthetic joint infections was considered an alternative treatment to prostheses removal and overall was well tolerated by patients [12].

## Conclusions

We describe a rare case of MRSA/MSSA meningitis in the setting of a recently placed brain graft. Guideline-directed therapy in this scenario is not well established. Our decision to treat the patient with oxacillin, vancomycin, and rifampin for 10 weeks was based on their synergistic effects and high rates of remission when used in conjunction. We opted to treat the patient with cefadroxil for suppressive therapy upon completion of the IV antibiotics, as this medication has been shown to have great CSF penetration.

Our case highlights the importance of prompt recognition and treatment of MRSA/MSSA meningitis due to the high morbidity associated with this disease. As there are no current guidelines discussing the management of MRSA/MSSA meningitis in the setting of a recently placed brain graft, our patient was treated with 10 weeks of IV antibiotics followed by indefinite suppressive therapy with cefadroxil. This patient showed significant improvement with this regimen, as her subsequent lumbar punctures indicated clearance of MRSA/MSSA from the CSF. In conclusion, MSSA meningitis should be considered as a differential diagnosis for meningitis in patients with recently placed brain grafts. Treatment should be geared towards susceptibilities, and we found that 10 weeks of IV antibiotics followed by indefinite cefadroxil significantly improved clearance of MRSA/MSSA meningitis.
